# Isolated Breast Relapse Mimicking Breast Cancer in Elderly Patient with Acute Lymphoblastic Leukemia

**DOI:** 10.4274/tjh.2013.0270

**Published:** 2014-06-10

**Authors:** Ajay Gogia, Prashant Mehta, Raja Pramanik, Rajive Kumar

**Affiliations:** 1 Dr. B.R.A. Institute Rotary Cancer Hospital All India Institute of Medical Sciences, Department of Medical Oncology, New Delhi, India; 2 Dr. B.R.A. Institute Rotary Cancer Hospital All India Institute of Medical Sciences, Lab of Oncology, New Delhi, India

**Keywords:** Acute lymphoblastic leukemia, Isolated breast relapse

## TO THE EDITOR

Acute lymphoblastic leukemia (ALL) in adults is associated with high relapse rates. Isolated extramedullary relapse other than in the central nervous system is rare in adult females with ALL. We present a case of isolated breast relapse in a 65-year-old female with ALL, mimicking breast cancer.

**Figure 1:**
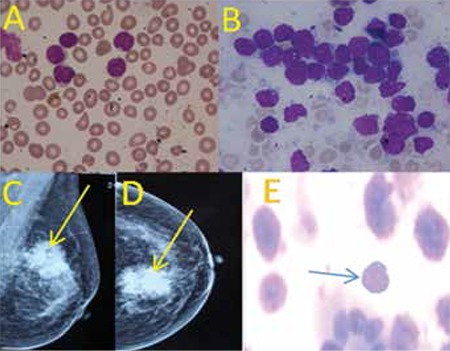
Peripheral blood smear and bone marrow aspirate(A&B) show lymphoblasts with coarse chromatin and highnuclear cytoplasmic ratio (Jenner-Giemsa stain, 1000x).Mediolateral oblique and cranio-caudal views of themammogram show homogeneous mass with architecturaldistortion and microcalcification (C&D). Fine-needleaspiration shows immature lymphoid cell with duct (400X)(E).

A 65-year old female presented with a 2-monthhistory of fever and generalized lymphadenopathy inDecember 2006. Physical examination revealed generalizedlymphadenopathy and hepatosplenomegaly. Her hemoglobinwas 11.9 g/dL, total leukocyte count 9.2x10^9^/L, and plateletcount 106x10^9^/L. Peripheral blood smear showed 5%myeloperoxidase-negative blast cells and the bone marrowrevealed near total replacement with lymphoblasts (Figures1A and 1B), which on flow cytometry were positive forCD19 and CD22 and negative for CD10, CD5, and CD7.The cerebrospinal fluid was uninvolved. She was diagnosedwith pre-B ALL and received induction as per the MCP-841protocol [1]. Remission was achieved at the end of inductionchemotherapy. Thereafter, she completed intensification,followed by oral maintenance for 1.5 years. Twenty-fourmonths later, she presented with a lump in the left breast.On clinical examination, she had a large, non-tender massin her left breast with a 1-cm mobile axillary lymph nodeon the same side. Systemic examination was normal. Weconsidered a diagnosis of breast cancer in view of the classical presentation, supported by a mammogram finding suggestiveof Breast Imaging Reporting and Data System Score V (Figures1C and 1D). Blood counts, peripheral blood smear, and bone marrow were unremarkable. Fine-needle aspiration of thebreast lump and axillary lymph node showed a monomorphicpopulation of immature cells with a high nuclear/cytoplasmicratio and prominent nucleoli, suggestive of leukemic blasts(Figure 1E). The blasts were positive for CD45, CD19, andCD22 and negative for CD3, CD5, CD7, CD10, and MPO onflow cytometry. A diagnosis of ALL relapse was made. Furtherinvestigations failed to reveal any other extramedullary siteof involvement. She was given reinduction using the sametreatment protocol. Response assessment done at the end ofinduction therapy showed complete resolution of the breastlump. The patient has been asymptomatic and disease-free forthe last 2 years. Informed consent was obtained.

The most common sites of relapse in ALL are the marrow,the central nervous system, and occasionally the ovaries.Breast involvement is not so rare in ALL but is usually seenin teenagers and is generally associated with the disease atother extramedullary sites as well as the bone marrow [2].The published literature revealed a total of 14 cases of isolatedbreast relapse in ALL. The median age in those patients was16 years [3]. Four cases with similar presentation of adultALL have been published (Table 1) [4,5,6,7]. Sagar et al.reported a similar occurrence in a 35-year-old female, butdetails on immunophenotyping, response, remission status,and outcome were not available [4]. Chim et al. reported asimilar case in a 23-year-old male with previous diagnosis ofT-cell ALL who died of progressive disease after 7 months ofrelapse [5]. Two other cases with similar presentation havebeen reported [6,7]. All patients reported with adult ALLwere between the ages of 23 and 35 years. Our patient was69 years old at the time of relapse, and her clinical findingsclosely resembled breast cancer, a malignancy more commonfor her age. It is important that clinicians be aware that thebreast may be a site of relapse in adult females with ALL andhence include breast examination during follow-up.

## CONFLICT OF INTEREST STATEMENT

The authors of this paper have no conflicts of interest, including specific financial interests, relationships, and/ or affiliations relevant to the subject matter or materials included.

## Figures and Tables

**Table 1:**
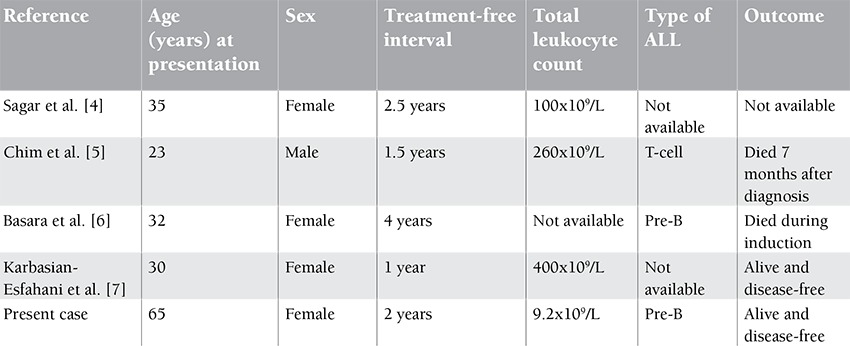
Cases of isolated breast relapse in adult ALL.
